# Long-Term Outcomes of Revisional Malabsorptive Bariatric Surgery: Do the Benefits Outweigh the Risk?

**DOI:** 10.1007/s11695-022-06019-7

**Published:** 2022-03-30

**Authors:** Kaleb Lourensz, Irsa Himantoko, Kalai Shaw, Cheryl Laurie, Louise Becroft, Edward Forrest, Peter Nottle, Daniel Fineberg, Paul Burton, Wendy Brown

**Affiliations:** 1 Department of Surgery, Central Clinical School, Monash University, Level 6, Alfred Centre, 99 Commercial Road, 3002 VIC Melbourne, Australia; 2 Oesophago-Gastric and Bariatric Surgical Unit, Department of General Surgery, The Alfred Hospital, Melbourne, 3004 Australia

## Abstract

**Purpose:**

To evaluate the long-term outcomes of revisional malabsorptive bariatric surgery.

**Materials and Methods:**

Malabsorptive bariatric procedures are increasingly performed in the revisional setting. We collated and analysed prospectively recorded data for all patients who underwent a revisional Biliopancreatic diversion + / − duodenal switch (BPD + / − DS) over a 17-year period.

**Results:**

We identified 102 patients who underwent a revisional BPD + / − DS. Median follow-up was 7 years (range 1–17). There were 21 (20.6%) patients permanently lost to follow-up at a median of 5 years postoperatively. Mean total weight loss since the revisional procedure of 22.7% (SD 13.4), 20.1% (SD 10.5) and 17.6% (SD 5.5) was recorded at 5, 10 and 15 years respectively. At the time of revisional surgery, 23 (22.5%) patients had diabetes and 16 (15.7%) had hypercholesterolaemia with remission of these occurring in 20 (87%) and 7 (44%) patients respectively. Nutritional deficiencies occurred in 82 (80.4%) patients, with 10 (9.8%) patients having severe deficiencies requiring periods of parenteral nutrition. Seven (6.9%) patients required limb lengthening or reversal procedures. There were 16 (15.7%) patients who experienced a complication within 30 days, including 3 (2.9%) anastomotic leaks. Surgery was required in 42 (41.2%) patients for late complications.

**Conclusion:**

Revisional malabsorptive bariatric surgery induces significant long-term weight loss and comorbidity resolution. High rates of temporary and permanent attrition from follow-up are of major concern, given the high prevalence of nutritional deficiencies. These data question the long-term safety of malabsorptive bariatric procedures due to the inability to ensure compliance with nutritional supplementation and long-term follow-up requirements.

**Graphical abstract:**

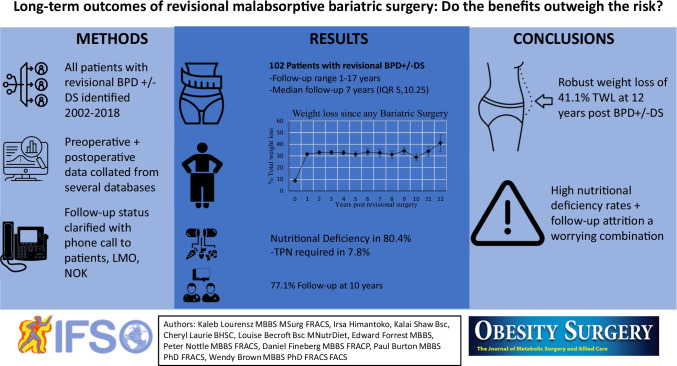

**Key points:**

• Revisional bariatric surgery workload is increasing

• Revisional malabsorptive surgery is efficacious for weight loss and comorbidity resolution

• Revisional malabsorptive surgery is associated with high rates of nutritional deficiencies

• Attrition from follow-up in this specific cohort of patients is of particular concern due to the risk of undiagnosed and untreated nutritional deficiencies

**Supplementary Information:**

The online version contains supplementary material available at 10.1007/s11695-022-06019-7.

## Introduction

Originally described in 1976 by Scopinaro, biliopancreatic diversion (BPD) is a form of resectional gastric bypass with a Roux-en-Y reconstruction. It has been shown to provide substantial weight loss and metabolic comorbidity resolution [1]. This procedure was subsequently modified by Hess, exchanging the distal gastrectomy for a sleeve gastrectomy known as the ‘Duodenal Switch’ (DS; BPD + DS) thus preserving the vagus nerve and the pylorus, aiming to reduce functional gut side effects [2, 3].

Multiple studies have demonstrated the efficacy of BPD and BPD + DS as a primary metabolic/bariatric procedure [4–8]. Despite this, malabsorptive surgery is rarely performed as a primary procedure in the modern era. In the most recent International Federation for the Surgery of Obesity and Metabolic disorders (IFSO), global survey of metabolic/bariatric surgical procedures, BPD and BPD + DS accounted for just 0.5% of all procedures performed worldwide [9]. This is possibly because of the nutritional deficiencies and complications that are commonly associated with these procedures [10, 11].

Weight recidivism, inadequate comorbidity resolution and complications such as reflux mean that a significant number of people with obesity who undergo a bariatric operation will need more than one procedure in their lifetime [11, 12]. Efficacy and safety benchmarks for revisional procedures need to be established to enable decision making regarding the most appropriate, safe and effective revisional procedures for the individual.

Ideally, a revisional procedure should have a different mechanism of action by which satiety and satiation are achieved when compared to the primary procedure. This may provide the patient with the best opportunity for resolution of metabolic comorbidities and reinstitution of weight loss. On this basis, malabsorptive procedures such as single anastomosis duodenal-ileal bypass with sleeve gastrectomy/one anastomosis duodenal switch (SADI-S/OADS) have become increasingly popular as revisional procedures following primary operations such as laparoscopic adjustable gastric band (LAGB)/sleeve gastrectomy (LSG)/gastroplasty [13].

From a technical point of view, a standard BPD also allows the surgeon to avoid the upper stomach that maybe scarred or distorted from the primary restrictive procedure, theoretically reducing the risk of leaks at the time of the revisional procedure when compared to procedures that require the angle of His to be dissected out such as in Roux-en-Y gastric bypass (RYGB).

Currently, there are no large long-term studies looking at efficacy (weight loss and comorbidity resolution), perioperative safety, risk of nutritional deficiencies or attrition from follow-up after malabsorptive procedures such as BPD and BPD + DS when performed as revisional procedures.

We undertook a retrospective longitudinal study to assess the long-term safety and efficacy of these procedures in the revisional setting.

## Methods

Ethics approval was obtained from the Cabrini Hospital and Alfred Hospital ethics committees, issue number 03–05-10–15 and 370/15 respectively.

### Perioperative Details

Patients who underwent a BPD or BPD + DS as a revisional procedure between the years 2002 and 2018 were included in this study.

Patient characteristics, past operative details, complications, follow-up status, weight and nutritional markers were collated from several databases used by the surgeon’s rooms or bariatric outpatient clinics. These databases included a separately maintained, custom-designed Microsoft Access (Microsoft Corp, Redmond, WA, USA) database, PowerChart (Cerner, North Kansas City, Missouri, USA) and Genie (Genie Solutions, Fortitude Valley, QLD, Australia).

### Outcomes

The primary outcome was diagnosis of a nutritional deficiency. This included major nutritional deficiency requiring surgical reversal/limb lengthening procedures, and minor deficiencies that were diagnosed via pathology testing and required specific additional oral supplementation or infusion.

Secondary outcomes include patients lost to follow-up, weight loss and post-operative complications.

### Local Indications for Revisional Malabsorptive Surgery

1. Inadequate weight loss or weight regain after primary bariatric procedure
2. Previous eroded gastric band
3. Previous gastroplasty
4. Demonstrated compliance with follow-up after previous bariatric surgery
5. Agreement to attend lifelong post-operative follow-up

Prior to surgery, all patients were educated on the need for life-long follow-up. This included dietician review focussed on nutritional counselling about dietary strategies after surgery and the need for lifelong vitamin supplementation.

### Operative Details

In our practice, BPD was not routinely used as a first-line revisional procedure and was recommended by the surgeon only when there was significant concern that other procedures may be precluded by the state of the upper stomach. A history of eroded gastric was considered an indication for a classical BPD to avoid dissection around the angle of His. Prior gastroplasty also was considered an indication for classical BPD, to avoid parallel staple lines in the upper stomach. All gastroplasties in our series were either the traditional vertical banded gastroplasty [14] or high gastric reduction [15]. Both techniques involve stapling through the fundus of the stomach without division. BPD + DS was more likely to be chosen when the patient had a prior sleeve gastrectomy, or the state of the upper stomach was better than expected.

The operative technique for BPD and BPD + DS follows the classical descriptions [1, 2]. Briefly, BPD involves formation of a 500-ml gastric pouch, resection of the distal stomach and closure of the duodenal stump. Gastrointestinal continuity is achieved by a Roux-en-Y reconstruction with a 250-cm alimentary limb and a 50-cm common channel, resulting in a long biliopancreatic limb. Over the years, however, the length of our common channels increased from 50 to 75 cm. BPD + DS involves the formation of a sleeve gastrectomy, division of the first part of the duodenum and Roux-en-Y reconstruction with a 250-cm alimentary limb anastomosed to the proximal part of the duodenum, and a 100-cm common channel. The laparoscopic procedure was performed in the lithotomy position, using the same principles outlined above. Six ports were used, including a Nathanson liver retractor for adequate exposure of the stomach. All anastomoses were handsewn with a 3.0 absorbable suture.

### Post-Operative Management

On discharge from hospital following surgery, all patients were counselled to follow a low fat, high protein diet and prescribed a bariatric specific multivitamin tablet along with a fat-soluble multivitamin supplement, providing 5000 IU of Vitamin A, 800 IU vitamin D and 105 mg of iron daily. Pancreatic lipase supplements and proton pump inhibitors were prescribed as required.

### Follow-up

Post-operative reviews occurred at 4 weeks, 4 months and then every 6 months, with dietician support being available at each review. Routine nutritional screening blood tests were completed prior to the 4-month review and then at 6-monthly intervals thereafter. These included a full blood examination, iron studies, albumin levels, zinc levels and vitamins A, D and B12 levels. Increased individual supplementation under close supervision was prescribed as required, with some patients requiring 50,000 IU of Vitamin A and 50,000 IU of Vitamin D daily for notable deficiencies.

Diabetes remission was defined as an HbA1c < 6.5% in the absence of any diabetic medications. Diabetes improvement was defined as an HbA1c < 6.5% on equivalent medications that the patient was on preoperatively. Hypercholesterolaemia remission was defined as total cholesterol < 5.2 mmol/L in the absence of any lipid lowering medications. Hypercholesterolaemia improvement was defined as a total cholesterol < 5.2 mmol/L whilst taking lipid lowering medications.

Early complications were defined as any deviation from normal post-surgical outcome within 30 days. Late complications were defined as any surgical complication requiring operative management, greater than 30 days from the time of surgery.

Active follow-up was defined as the patient having seen a medical practitioner for the purpose of bariatric surgical follow-up within the last 2 years. Contact was attempted with any patient who had not seen their surgeon in private rooms, or an associated public bariatric clinic within the last 2 years. A phone call was made to the patient, their next of kin and nominated general practitioner. If contact was unable to be made, or follow-up criteria unable to be met, patients were deemed permanently lost to follow-up. Temporary attrition from follow-up was defined as a patient in active follow-up, with greater than 18 months in between follow-up appointments.

### Data Analysis

Continuous data was presented as mean and standard deviation, or median and interquartile range. Kaplan–Meier survival analysis was used for determining the risk of developing nutritional deficiencies and attrition from follow-up. Patients who were confirmed to be deceased were ‘right censored’. Independent samples *t* test was used to compare means of continuous, normally distributed data. Dichotomous data was analysed using the chi-squared test. Data was analysed using SPSS version 27 (SPSS Inc., Chicago, IL, USA).

## Results

### Baseline Characteristics

A total of 102 patients with a revisional BPD or BPD + DS were included in the study. Prior to this, all patients either had undergone one or more of the following procedures: gastric band placement or revision, sleeve gastrectomy or gastroplasty. Of the 45 patients with a prior gastroplasty procedure, 3 underwent a traditional vertical banded gastroplasty and 42 had a high gastric reduction. At the time of the primary bariatric procedure, median BMI was 49.3 kg/m^2^ and median weight was 130 kg. At the time of the revisional procedure, the median BMI was 41.3 kg/m^2^ and median weight was 120 kg (Table 1).

**Table 1:** Patient demographics

Variable	Value *n* = 102 *n* (%)
Gender	
Male	14 (13.7)
Female	88 (86.3)
Age	
Mean ± SD	49 ± 9.2
Range	25–64
Highest recorded weight, kg	
Median (IQR)	130 (120–154.3)
Range	90–237
Highest BMI, kg/m^2^	
Median (IQR) Range	49.3 (44.7–57.0) 36.0–80.1
Operative weight, kg	
Median (IQR)	120 (109–139)
Range	77–224
Operative BMI, kg/m^2^	
Median (IQR)	41.3 (40.9–51.3)
Range	25.7–75.0
Operative excess weight loss, %	
Median (IQR)	13.1 (0–26.6)
Range	0–95.1
Operative comorbidities	
Diabetes	24 (23.5)
Hypercholesterolemia	16 (15.7)
Previous bariatric surgery	
One prior procedure:	29 (28.4%)
Gastroplasty	20
Gastric Band	8
LSG	1
Two prior procedures:	30 (29.4%)
Gastric band procedures	19
Gastroplasty procedures	11
Three prior procedures:	18 (17.7%)
Gastric band procedures	11
Gastroplasty(s) + gastric band(s)	5
Gastroplasty procedures	2
Four + prior procedures:	25 (24.5%)
Gastric band procedures	9
Gastric band(s) + gastroplasty(s)	6
Gastroplasty(s) + LSG	1
Gastric band(s) + gastroplasty(s) + LSG	9
Total previous bariatric procedure	
Median (IQR)	2 (1–3)
Range	1–7

### BPD vs BPD + DS

Of the 102 patients, 77 had a classic BPD and 25 had a BPD + DS. There was no statistically significant difference in attrition from follow-up, major or minor nutritional deficiencies or weight loss at 1- or 5-year post-revisional procedure (Table 2). Only 1 patient with a BPD + DS was available for follow-up after 10 years. Given this, data was pooled between the two groups, and the rest of the analysis was performed as a single cohort (abbreviated BPD + / − DS).

**Table 2:** BPD vs BPD + DS

	BPD	BPD + DS	*p* value
*n*	77	25	
Mean TWL% 1 year	23.0	22.3	0.788
Mean TWL% 5 years	21.0	30.9	0.102
Mean follow-up (years)	8.6	9.4	0.477
Nutritional deficiency diagnosis (%)	61 (79.2)	21 (84)	0.514
Iron infusion (%)	31 (40.3)	5 (20)	0.066
Reversal/limb lengthening surgery (%)	4 (5.2)	3 (12)	0.358

### Previous Surgical Details

Before the revisional BPD + / − DS, 29 patients had 1 prior bariatric procedure, 30 patients had 2 prior bariatric procedures, 18 patients had 3 and 25 patients had 4 or more (Table 1). Eleven patients previously had an eroded gastric band.

### Perioperative Details

An open BPD + / − DS was performed in 98 patients, 3 were completed laparoscopically and 1 was commenced laparoscopically and subsequently converted to an open procedure.

There was no in-hospital mortality. There were 16 (15.7%) patients who experienced an early complication, requiring 7 (6.9%) patients to be readmitted to hospital.

Anastomotic leak occurred in 3 (2.9%) patients. Of these, 1 patient resolved with antibiotics and cessation of enteral feeds, 1 patient required percutaneous drainage and 1 patient required a return to theatre for washout and drainage.

There were 7 (6.9%) anastomotic strictures, all of which followed classic BPD, and were managed with endoscopic balloon dilatation.

Sixty-two late complications were recorded in 42 patients (41.2%) necessitating further surgery. Thirty-one (50%) of these complications were uncomplicated incisional hernias (Table 3).

**Table 3:** Post-operative details

Variable		*n* (%)
Hospital length of stay (days)		
Median (IQR)		8 (7–9)
Range		3–27
Unplanned readmission		7 (6.9)
Return to theatre		5 (4.9)
Unplanned ICU admission		1 (1)
Intra-operative mortality		0 (0)
In-hospital mortality		0 (0)
Intra-operative complication		0 (0)
< 30 days (early) complication		16 (15.7)
Anastomotic leak		3 (2.9)
Bleeding		1 (1)
Anastomosis stricture		3 (2.9)
Deep vein thrombosis		1 (1)
Wound infection		11 (10.8)
> 30 days (late) complication		42 (41.2)
Anastomosis stricture		4 (3.9)
Gastric stricture		1 (1)
Small-bowel obstruction		2 (2)
Gallstones		10 (9.8)
Uncomplicated incisional hernia		31 (30.4)
Poor weight loss		8 (7.8)
Severe malnutrition		6 (5.9)

### Follow-up

Length of follow-up ranged from 1 to 17 years with median follow-up of 7 (IQR 5, 10.25) years throughout the study period. Three patients died during the follow-up period for reasons unrelated to the BPD + / − DS procedure, resulting in 21 (20.6%) patients permanently lost to follow-up at a median of 5 years (IQR 3.5, 8.5).

At the time of data collection (August 2019), 45 (44.1%) patients were engaged in active follow-up with their treating team. Upon further contact, 33 (32.4%) patients were found to be engaging in follow-up with another service. Of the 78 (76.5%) patients in active follow-up, 42 (54%) infrequently attended follow-up visits, with at least one gap of greater than 18 months between appointments.

There were 86 patients eligible for review at 5 years, 48 patients at 10 years and 13 patients at 15 years. Follow-up was achieved in 79 (91.9%) at 5 years, 37 (77.1%) at 10 years and 10 (76.9%) patients at 15 years.

**Fig. 1:**
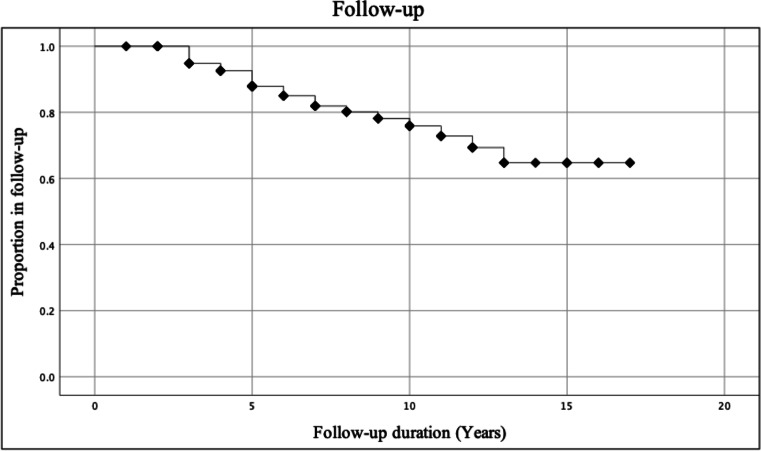
Risk of permanent attrition from follow-up at different time points using Kaplan–Meier survival analysis

Survival analysis demonstrates a steady decrease in the rate of follow-up to 13-year post-surgery, before plateauing. An estimated 64.7% of patients will remain in follow-up after 17 years (Fig. 1).

### Nutritional Deficiencies

**Fig. 2:**
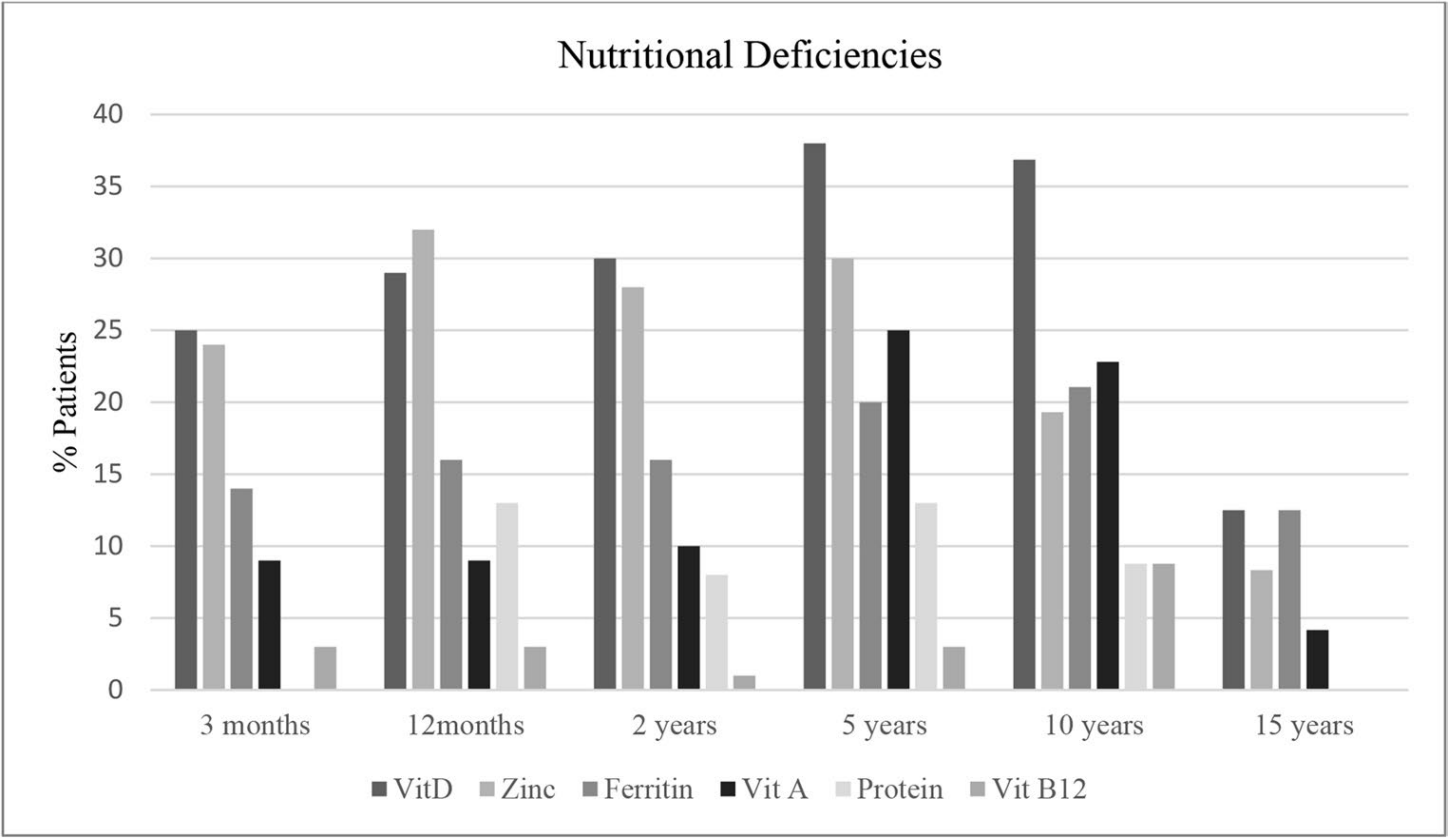
Comparison of the prevalence of nutritional deficiencies at different time points

Nutritional deficiencies were identified in 82 (80.4%) patients. Figure 2 demonstrates the point prevalence of individual nutritional deficiencies at various time points during the follow-up period. Vitamin D was the most common deficiency identified in 61 (59.8%) patients, followed by zinc in 53 (51.9%) patients and ferritin in 44 (43.1%) patients. Iron infusions were received by 36 (35%) patients. Severe nutritional deficiencies were identified in 10 (9.8%) patients, 8 of which required a period of hospitalisation and parenteral nutrition.

**Fig. 3:**
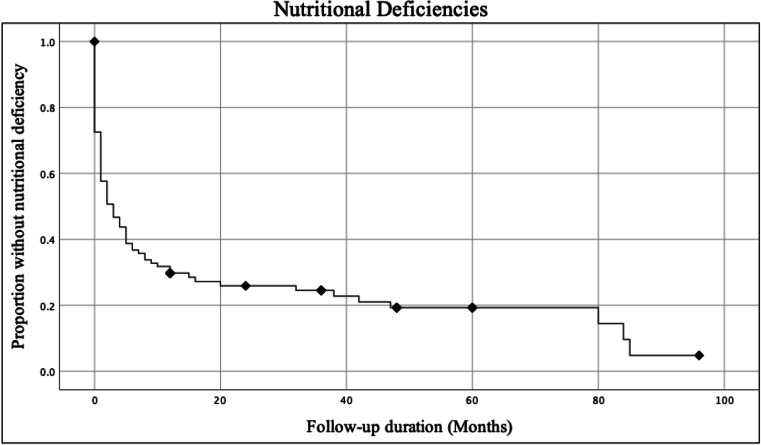
Risk of any nutritional deficiency being diagnosed at different time points using Kaplan–Meier survival analysis

Kaplan–Meier survival analysis (Fig. 3) estimates a median 3 months (95% CI 0.90 to 5.10) from the time of surgery to diagnosis with a nutritional deficiency, with over 95% of patients having a nutritional deficiency within 7 years of their revisional surgery.

### Surgical Revisions

Surgical reversal or common limb lengthening procedures were performed in 7 (6.9%) patients. This occurred at a median of 6.5 years (IQR 1.5, 9.5) after BPD + / − DS. Six of these were due to malnutrition, and 1 was reversed to treat severe diarrhoea. Four of these patients previously had a BPD, and 3 had a BPD + DS.

### Weight Loss

**Fig. 4:**
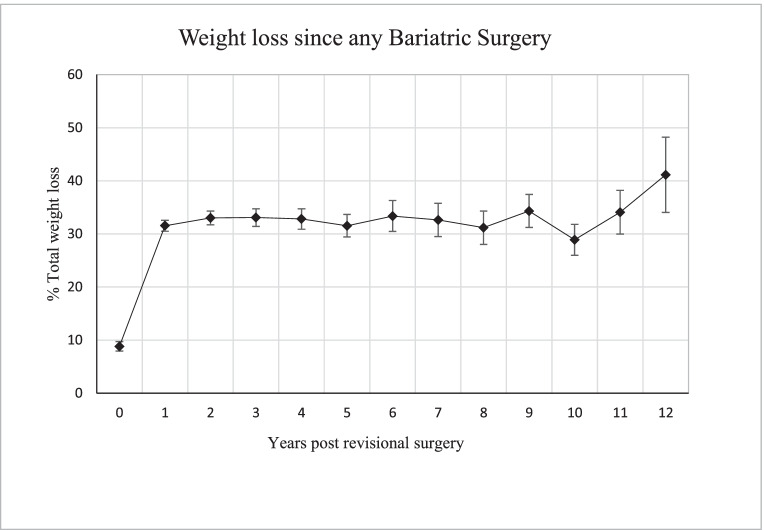
Mean weight loss over time + /** − **SEM. Data presented to 12 years due to less than 15 patients with longer follow-up

Cumulative long-term weight loss data post primary and revisional bariatric surgery is demonstrated in Fig. 4. Prior to the revisional surgery, patients had lost a mean of 12.6 kg (8.8% TWL) from their heaviest recorded weight prior to the primary procedure. Following revisional surgery, sustained weight loss was demonstrated throughout the 17-year period, with a further mean TWL of 22.7% (SD 13.4), 20.1% (SD 10.5) and 17.6% (SD 5.5) recorded at 5, 10 and 15 years respectively. Cumulatively, maximal mean weight loss of 47.6 kg (41.1% TWL) following any bariatric surgery was found at 12 years post BPD + / − DS.

### Comorbidities

At the time of revisional surgery, 23 (22.5%) patients had diabetes mellitus, and 16 (15.7%) patients had hypercholesterolaemia. Of these, 20 (87%) patients had documented remission of their diabetes during the post-operative period, and 7 (44%) patients had complete remission of their hypercholesterolaemia, with a further 7 (44%) showing an improvement in their hypercholesterolaemia during the follow-up period.

## Discussion

Whilst metabolic/bariatric surgery provides powerful weight loss and health benefits for most patients, the incidence of revisional metabolic/bariatric surgery for weight regain, comorbidity recurrence and functional side effects have been increasing. Malabsorptive procedures, such as BPD, BPD + DS and SADI-S/OADS, have been proposed as potential options for patients requiring revisional metabolic/bariatric surgery as they offer a different mechanism of action to commonly performed primary procedures such as LSG. However, there is currently a paucity of data describing the long-term outcomes of malabsorptive procedures in the revisional metabolic/bariatric surgical setting.

We have presented the first large comprehensive long-term study demonstrating the outcomes of malabsorptive surgery as a revisional metabolic/bariatric procedure. We have shown that whilst BPD + / − DS provides satisfactory long-term weight loss and resultant comorbidity resolution, this comes at the cost of significant surgical and nutritional complications.

Maximal weight loss was found 12 years post BPD + / − DS with a mean TWL of 32.3%. When combined with the mean TWL of 8.8% prior to the BPD + / − DS, our patients achieved over 40% TWL when compared with their baseline weight prior to any bariatric surgery. There was an apparent tapering of mean weight loss after 15 years. This likely relates to the small numbers of patients eligible for follow-up at this timepoint.

High rates of comorbidity resolution were achieved, demonstrating the metabolic efficacy of malabsorptive revisional surgery in patients with resistant diabetes mellitus or hypercholesterolaemia that had not resolved following the primary procedure.

Post-operative nutritional deficiencies, however, were found to be extremely common, with the incidence increasing throughout the follow-up period. Over 50% of patients in this study were diagnosed with a nutritional deficiency within the first 3 months of their revisional malabsorptive surgery, despite being discharged with appropriate vitamin supplementation. Severe malnutrition mandated surgery to reverse the malabsorption in 5.9% of patients.

Nutritional deficiencies are common prior to metabolic/bariatric surgery and are common after many other metabolic/bariatric procedures including LSG and RYGB [16, 17]. Adherence to long-term multivitamin supplementation is known to be poor following metabolic/bariatric surgery [18] although long-term follow-up studies have established that patients actively engaged with their specialist medical and allied health care providers are more likely to take vitamin supplements [19].

Although our overall follow-up rate compares favourably with the literature [11, 20, 21], it is of significant concern that in our cohort, there were 21 (20.6%) patients who were permanently lost to follow-up and a further 42 (41.2%) patients who had prolonged gaps in their follow-up. This occurred despite the importance of follow-up being emphasised in pre/post-operative consultations; for patients to be offered a BPD + / − DS, they needed to have previously demonstrated compliance with follow-up. The risk of significant undiagnosed and untreated nutritional deficiencies in these patients remains unacceptably high.

These procedures had a reasonable early perioperative safety profile, with only 2.9% of patients having an anastomotic leak. This is an improvement on the other published rates of anastomotic or staple-line leak following revisional BPD + / − DS of 5.1–11.6% [22–24]. RYGB has been reported as having a leak rate of 2.7% in the revisional setting [25]. In the setting of an eroded gastric band (as was the case with 11 of our patients), revisional surgery can be high risk and technically challenging. With modern technique and expertise, procedures such as the laparoscopic RYGB (when possible) may be a more feasible option in the present era and avoid the attendant risks and consequences of BPD.

The strengths of this study are the length of follow-up achieved, as well as a unique focus on nutritional deficiencies and follow-up attrition rates as outcome measures. Limitations inherent to this type of retrospective study include selection bias. BPD + / − DS was only offered on an individual case-by-case basis, where the surgeon felt it was indicated and the patient was accepting. As such, only principles guiding the decision for BPD or BPD + DS could be discussed. Additionally, the absolute numbers of patients with a BPD + DS were relatively small, meaning any subgroup analysis was underpowered to look for differences between the two groups. With only 38 patients at 10 years and 13 patients at 15 years available for follow-up, there are significantly diminished numbers of patients eligible for follow-up at the latter end of the review period. Limited data on comorbidities was maintained prospectively on the database. Diabetes and hypercholesterolaemia were selected to represent a change in metabolic comorbidity status. A more comprehensive evaluation for a range of comorbidities would be of significant value in a future undertaking. Although we went to great effort to comprehensively track patients being followed up externally and define whether they were genuinely lost to follow-up, it simply is not possible to be certain patients are not being followed up elsewhere. This could lead to overestimating rates of attrition from follow-up and underestimating nutritional deficiencies.

Although 42 (41.2%) patients experienced a late complication requiring 62 operations, half of these procedures were uncomplicated incisional hernia repairs. Ninety-eight (96.1%) of these procedures were commenced via laparotomy; practice has changed over the course of this longitudinal study, and it is hoped that the shift to laparoscopic surgery will reduce the risk of this complication in the future. This is also a potential limitation of the study, as the change to laparoscopic operating may also affect other outcomes. However, we feel the main outcomes of this study—robust weight loss, common nutritional deficiencies and attrition from follow-up—are unlikely to be meaningfully changed as a result.

Future studies need to compare the safety and efficacy of procedures that are considered to incorporate a malabsorptive element in the revisional setting. Better mechanisms for reducing follow-up attrition and compliance with nutritional supplementation are required. This is of particular importance as revisional procedures such as SADI-S/OADS are being performed when long-term metabolic and nutritional outcomes have yet to be elucidated. Other areas of focus should be the long-term impact of these procedures on clinical parameters such as bone density and osteoporosis rates.

## Conclusion

Revisional malabsorptive metabolic/bariatric surgery induces long-term weight loss and comorbidity resolution at the expense of significant long-term nutritional deficiencies. Long-term follow-up is essential to identify and treat nutritional deficiencies; however, temporary or permanent attrition from follow-up interrupts this process in the majority of patients. A more comprehensive understanding of the risks and benefits is required prior to primarily malabsorptive procedures being generally recommended as a revisional metabolic/bariatric surgical option.

## Supplementary Information

Below is the link to the electronic supplementary material.

Supplementary file1 (DOCX 14 kb)
