# Supplementary material for: Long-Term Outcomes of Revisional Malabsorptive Bariatric Surgery: Do the Benefits Outweigh the Risk?
Source: Obes Surg. 2022 Mar 30;32(6):1822–30. doi: 10.1007/s11695-022-06019-7 (PMC9072481; doi:10.1007/s11695-022-06019-7)
Supplement: Supplementary file 1 — Supplementary file1 (DOCX 14 kb) [file 11695_2022_6019_MOESM1_ESM.docx]

| **Nutritional Marker** | **Low normal range** | **Unit** |
| --- | --- | --- |
| Albumin | 33 | g/L |
| Vit A | 1.0 | mcmol/L |
| Vit D 3, 25 | <50 | nmol/L |
| B12 | 138 | pmol/L |
| Serum Iron | 7 | mcmol/L |
| Ferritin | 30 | mcg/L |
| Zinc | 12.0 | mcmol/L |
